# Non-Intrusive Load Monitoring Based on Multi-Feature Fusion and Combinatorial Optimization Networks

**DOI:** 10.3390/s26154752

**Published:** 2026-07-27

**Authors:** Yubo Wang, Shuai Zhang, Zhiyou Cheng

**Affiliations:** 1 School of Internet, Anhui University, Hefei 230039, China; yubowang28@gmail.com; 2 School of Electronics and Information Engineering, Anhui University, Hefei 230601, China; p24101008@stu.ahu.edu.cn

**Keywords:** Non-Intrusive Load Monitoring (NILM), Particle Swarm Optimization (PSO), combinatorial optimization network, multi-feature fusion, conductance-time trajectory, mean-square current color-block map

## Abstract

To address the limitations of traditional Voltage-Current (VI) trajectory features in appliance load identification—such as the difficulty in distinguishing similar appliances, weakened amplitude information, and the absence of dynamic characteristics—this paper proposes a dual-stage cyclic training method for load identification that integrates multi-feature reconstruction with Particle Swarm Optimization (PSO). First, to overcome the high similarity of original VI trajectories, a PSO-based threshold optimization algorithm is designed to reconstruct VI trajectories through reflection operations and normalization, thereby enhancing the geometric morphological differences among similar appliances. Second, to supplement dynamic impedance information and energy level features, conductance-time trajectories and mean-square current color-block maps are introduced to characterize dynamic impedance variations and energy level differences, respectively. Finally, a three-channel classification network based on ResNet18 is constructed, where the reconstructed VI trajectories, conductance-time trajectories, and mean-square current color-block maps are fused via RGB channels as inputs, forming a closed-loop “threshold optimization—feature reconstruction—cyclic training” framework. Experimental results on the PLAID dataset demonstrate that the proposed method achieves an identification accuracy of 98.29% and a macro-averaged F1-score of 97.93%. Comparative experiments verify the effectiveness of the reconstructed VI trajectories, the complementarity of multi-feature fusion, and the superiority of the combinatorial optimization network, significantly improving the identification of multi-state and similar-condition appliances.

## 1. Introduction

With the continuous expansion of power systems and the rapid advancement of smart grid infrastructures, increasing demands have been placed on fine-grained energy management and load-side observability. In recent years, under the strategic framework of carbon neutrality and emission reduction targets, enhancing energy efficiency, optimizing load structures, and promoting energy-saving awareness among end users have become central priorities in modern power system development. Conventional energy monitoring approaches typically rely on deploying dedicated sensors on individual appliances to measure their power consumption. However, such intrusive solutions are costly and difficult to scale in large-scale practical applications.

In contrast, Non-Intrusive Load Monitoring (NILM) enables appliance-level energy disaggregation by acquiring aggregate voltage and current signals at a single measurement point and decomposing them into individual appliance operations through advanced signal processing and learning algorithms. As summarized in prior NILM surveys, this non-intrusive sensing paradigm eliminates the need for additional hardware deployment while providing fine-grained consumption insights for smart homes, energy management systems, demand response, and intelligent power grids [1].

Early studies on NILM primarily relied on handcrafted features and traditional machine learning techniques, such as those based on power variations, harmonic components, and transient signatures. Although these methods achieved reasonable performance under controlled conditions, they are heavily dependent on expert knowledge and often suffer from limited generalization capability in complex environments or when the number of appliance types increases. With the rapid development of deep learning, neural NILM models based on feed-forward networks, recurrent LSTM structures, and sequence-to-point learning have been increasingly adopted for energy disaggregation [2,3,4]. Residual CNN backbones further provide an effective basis for image-based feature recognition tasks [5]. To further enhance feature extraction and temporal modeling, recent advancements have pushed the boundaries of neural NILM by incorporating more sophisticated architectures. For instance, deep convolutional neural networks combined with load trajectories and multi-feature fusion have demonstrated superior discriminative capabilities [6]. Building upon this, cutting-edge multi-stream networks have been recently proposed to fuse multimodal sequential and image features, utilizing interpretable screening mechanisms to effectively reduce redundancy and mitigate inter-class load confusion in complex scenarios [7]. Concurrently, transformer-based attention mechanisms have been introduced to capture long-range temporal dependencies more effectively [8], while multi-appliance-task learning frameworks have been proposed to address the challenge of limited labeled data in practical scenarios [9]. Public datasets such as PLAID have also promoted the reproducible evaluation of these advanced load identification methods [10].

Within this paradigm, a critical challenge lies in how to effectively transform raw electrical signals into representations that are well-suited for deep learning models. Feature-engineering studies have explored binary VI trajectory mapping, low-frequency spectral signatures, and VI-trajectory-based load monitoring algorithms to capture appliance-specific electrical behavior [11,12,13]. VI trajectory images have also been combined with CNNs for appliance classification [14]. Furthermore, to mitigate the loss of magnitude and temporal characteristics during standard trajectory pixelation, recent state-of-the-art frameworks have introduced visualized color V-I trajectories combined with self-supervised contrastive learning and adversarial domain adaptation to effectively align cross-domain feature distributions [15]. In parallel, denoising autoencoders and transient-signal representations have been used to improve NILM robustness under different sampling conditions [16,17]. Hybrid ANN-PSO frameworks and Siamese-network identification further show the value of optimization and metric-learning strategies in NILM [18,19,20].

However, existing VI-based methodologies encounter three critical bottlenecks in practical complex scenarios. First, appliances with similar internal topologies (e.g., heating devices) exhibit highly analogous VI trajectories, making it difficult to distinguish them purely by geometric shape. Second, the standard normalization process in VI trajectory mapping inherently suppresses amplitude information, which is a vital discriminative factor for differentiating appliances of the same type but with distinct power ratings. Third, static VI trajectories fail to capture the transient and dynamic impedance variations that occur during appliance operation. Due to these unresolved challenges, readers and practitioners are often limited by the constrained discriminative power of traditional methods.

To bridge this research gap and overcome the aforementioned bottlenecks, this paper proposes a dual-stage cyclic training method for load identification that integrates multi-feature reconstruction with Particle Swarm Optimization (PSO). The main contributions of this study to the NILM and machine learning literature are threefold:

1. Novel VI Trajectory Reconstruction Methodology: We propose a threshold-based reflection transformation algorithm optimized by PSO. This explicitly disrupts the high structural similarity of original VI trajectories, enhancing the geometric and morphological differences among appliances with analogous operations.
2. Dynamic and Energy-Level Feature Augmentation: To resolve the issues of weakened amplitude information and the absence of dynamic characteristics in traditional VI images, we introduce two complementary features: the conductance-time trajectory (capturing dynamic impedance) and the Current-Squared Energy Colormap (capturing global energy differences).
3. Closed-loop Combinatorial Optimization Framework: We design an integrated deep learning architecture that fuses the three constructed features via RGB channels and continuously refines the trajectory thresholds through a “threshold optimization—feature reconstruction—cyclic training” closed loop, offering a new paradigm for adaptive feature engineering in NILM.

## 2. Reconstructed VI Trajectory

Existing studies typically categorize household appliances into several fundamental types, including purely resistive loads, reactive loads, loads without power factor correction, loads with power factor correction, and complex or hybrid-structure loads. Appliances within the same category generally exhibit highly similar electrical characteristics, particularly in their current trajectories. Event-based recognition and scalable real-time NILM systems have also shown that such similarities complicate robust appliance discrimination in practical settings [21,22]. During steady-state operation, although the supply voltage may exhibit minor fluctuations, it remains relatively stable within a standard range. Consequently, accurate load characterization requires jointly analyzing both voltage and current signals, as current variations are inherently influenced by voltage dynamics.

A widely adopted approach is to map single-cycle voltage and current measurements onto a two-dimensional plane to construct voltage–current (VI) trajectory images. This representation has demonstrated strong capability in capturing appliance-specific electrical features and has been extensively used in NILM tasks [23]. Real-time simultaneous-switching monitoring, empirical VI-signature analysis, and embedded low-sampling-rate identification further indicate that VI-based features must retain both morphology and amplitude-related information [24,25,26]. However, despite its effectiveness, the VI trajectory representation suffers from inherent limitations. For appliances belonging to the same fundamental category, their VI trajectories tend to exhibit high structural similarity, which significantly increases the difficulty of discrimination. This issue becomes more pronounced when differences primarily lie in current amplitude rather than waveform shape. In such cases, even when the original current waveforms differ considerably within a cycle, the resulting VI trajectories may still appear nearly indistinguishable after mapping. This phenomenon arises because many appliances share similar internal components and operating principles, leading to comparable waveform patterns. As a result, without explicitly incorporating amplitude-related information from both voltage and current signals, it remains challenging to achieve reliable appliance-level discrimination. To address these challenges, this study proposes a reconstructed VI trajectory method, which enhances the discriminative capability of traditional VI representations by transforming the original voltage–current mapping through a novel reconstruction strategy.

### 2.1. Reconstruction Algorithm

After the appliance reaches a steady operating state and remains stable for a sufficient duration, multiple single-cycle voltage and current sequences, denoted as *v* and *i*, are collected according to the condition defined in (1).

(1) $$v_{k}\times v_{k-1}<0\wedge v_{k-1}<0$$

Using the acquired raw data, the original voltage–current (VI) trajectory is first constructed. Subsequently, the trajectory is reconstructed by applying a threshold-based reflection transformation to both voltage and current signals, as described in (2)–(7).

(2) $$\Delta v=v_{k}-v_{\mathrm{TD}}$$

(3) $$\begin{array}{cc} \; & \left\{\begin{array}{c} f_{k}=\left\lfloor \frac{\Delta v}{2\times v_{\mathrm{TD}}}\right\rfloor \\ r_{k}=\Delta v\%\left(2\times v_{\mathrm{TD}}\right)\\ o_{k}=f_{k}\%2 \end{array}\right. \end{array}$$

(4) $$\begin{array}{cc} v_{k}^{\prime }= & \begin{array}{c} \left(2\times \mathrm{sign}\left(o_{k}\right)-1\right)\times \mathrm{sign}\left(v_{k}\right)\\ \;\times \;r_{k}-v_{\mathrm{TD}}\times \mathrm{sign}\left(v_{k}\right) \end{array} \end{array}$$

(5) $$\Delta i=i_{k}-i_{\mathrm{TD}}$$

(6) $$\begin{array}{cc} \; & \left\{\begin{array}{c} f_{k}^{\prime }=\left\lfloor \frac{\Delta i}{2\times i_{\mathrm{TD}}}\right\rfloor \\ r_{k}^{\prime }=\Delta i\%\left(2\times i_{\mathrm{TD}}\right)\\ o_{k}^{\prime }=f_{k}^{\prime }\%2 \end{array}\right. \end{array}$$

(7) $$\begin{array}{cc} i_{k}^{\prime }= & \begin{array}{c} \left(2\times \mathrm{sign}\left(o_{k}^{\prime }\right)-1\right)\times \mathrm{sign}\left(i_{k}\right)\\ \;\times \;r_{k}^{\prime }-i_{\mathrm{TD}}\times \mathrm{sign}\left(i_{k}\right) \end{array} \end{array}$$

In these equations, the operator % denotes the remainder operation, and ⌊·⌋ represents the floor operator. The variables $v_{k}$ and $i_{k}$ denote the *k*-th sample point of the voltage and current sequences within a power-frequency cycle, respectively. The parameters $v_{TD}$ and $i_{TD}$ represent the predefined voltage and current thresholds, while $v_{k}^{\prime }$ and $i_{k}^{\prime }$ denote the reconstructed voltage and current values after transformation. Following reconstruction, the transformed voltage and current sequences are normalized to ensure that their values fall within a unified range, thereby facilitating consistent feature representation. Based on the reconstructed and normalized sequences, a VI trajectory image is generated using an RGB color encoding scheme. Specifically, the voltage sequence is mapped to the vertical axis, and the current sequence is mapped to the horizontal axis. The trajectory is rendered in red (RGB: 255, 0, 0), while the background is set to black (RGB: 0, 0, 0), resulting in a visually structured representation suitable for subsequent input into deep learning models.

### 2.2. Threshold Optimization Based on Particle Swarm Optimization

The proposed reconstruction method relies on threshold-based reflection applied to voltage and current signals. The selection of these thresholds plays a critical role in determining the quality of the reconstructed VI trajectories. Specifically, excessively large thresholds may lead to over-compression along the current axis, resulting in the loss of fine-grained electrical characteristics, whereas overly small thresholds may introduce excessive reflection operations, causing structural distortion of the trajectory. Both scenarios can significantly degrade the performance of the appliance identification model. Therefore, determining an optimal pair of voltage and current thresholds is essential for maximizing classification accuracy.

**Figure 1:**
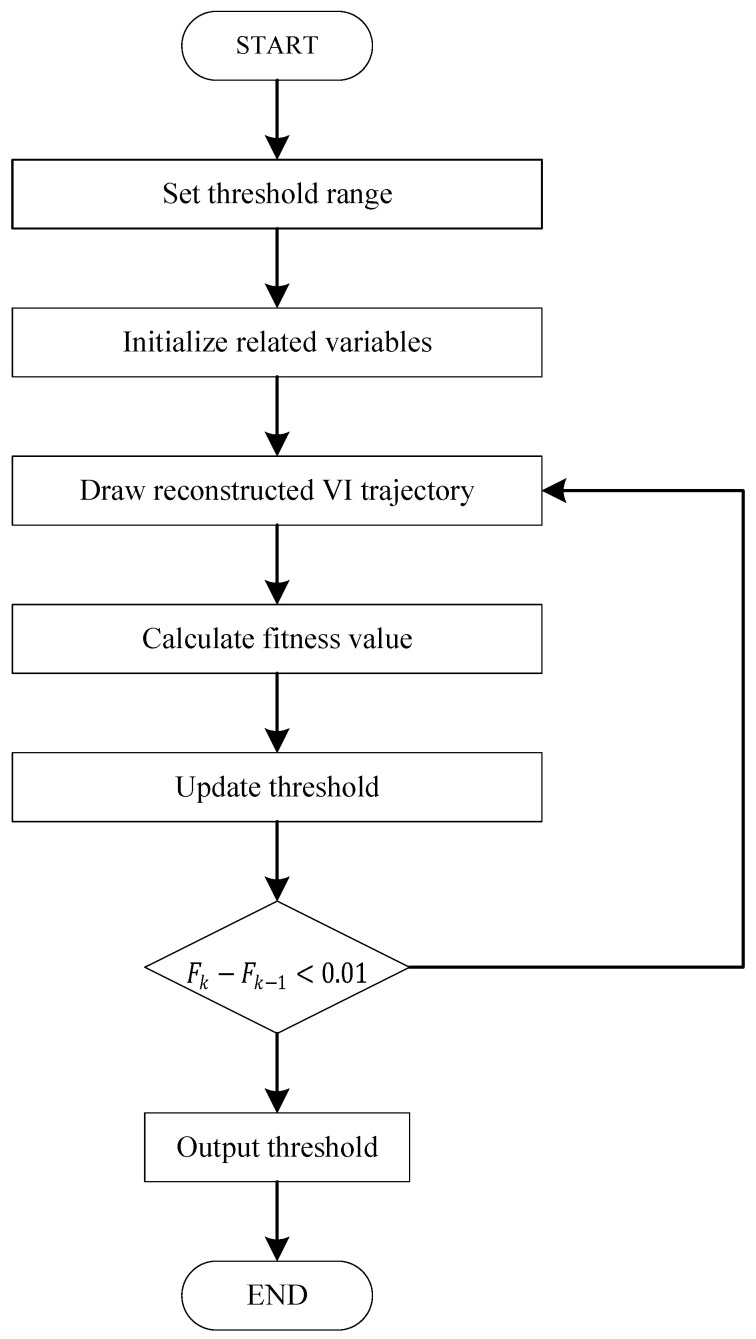
Flowchart of the PSO-based threshold optimization procedure.

To achieve this in an efficient and adaptive manner, a Particle Swarm Optimization (PSO) algorithm is employed, as illustrated in Figure 1 [20]. PSO is a population-based optimization technique that iteratively searches for the optimal solution by simulating the collective behavior of particles. Each particle represents a candidate solution in the search space, and its quality is evaluated using a predefined fitness function. During the optimization process, particles update their positions and velocities based on their historical best positions and the global best solution, exhibiting a tendency to converge toward the optimal region through cooperation and competition. The PSO-based optimization procedure for voltage and current thresholds is summarized as follows:

1. The search ranges for the voltage and current thresholds are defined based on practical operating conditions of household appliances. Specifically, the voltage threshold is constrained within 0∼350 V, while the current threshold is set within 0∼10 A.
2. Initialize the swarm, including particle positions, velocities, inertia weights, individual best positions, and the global best position.
3. The fitness function is defined as in (8), which jointly considers model performance and computational efficiency
  (8) F=a(1−Acc)×(bL)×(cT)
  The fitness function adopts a multiplicative structure to simultaneously minimize classification error (1−Acc), reconstruction loss (L), and computational time (T). Following the established PSO optimization framework for V-I trajectory reconstruction [23], the multiplicative penalty is utilized to strictly avoid sub-optimal solutions where any single metric performs poorly. Furthermore, the coefficients *a*, *b*, and *c* (constrained by a+b+c=1) act as attention weights that reflect the empirical “degree of attention” assigned to each attribute. This allows the model to establish a clear priority hierarchy (e.g., prioritizing accuracy over optimization time) within the multiplicative optimization space.
4. Update the voltage and current thresholds according to the particle positions, and reconstruct the VI trajectory images using the updated thresholds.
5. Compute the fitness value for each particle based on the reconstructed VI images and corresponding model performance.
6. Update particle velocities and positions, as well as individual and global optimal solutions, according to the PSO update rules.
7. Evaluate the stopping criterion. If the condition is not satisfied, return to Step 2 for further iterations; otherwise, output the optimal voltage and current thresholds as the final solution.

### 2.3. Reconstructed VI Trajectory Visualization

**Figure 2:**
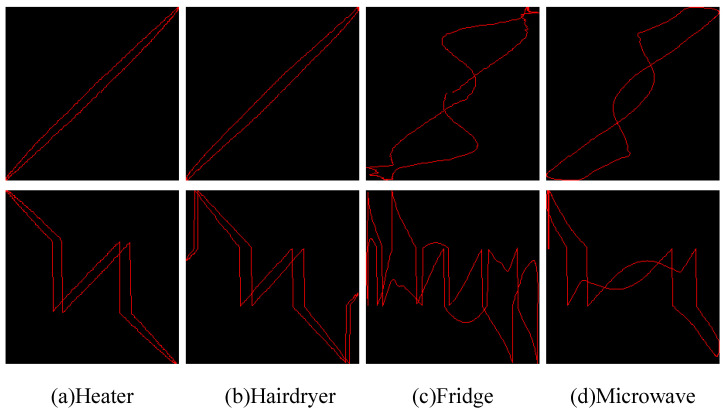
Original VI images (**top**) and reconstructed VI images (**bottom**) for representative appliances.

The VI trajectory images obtained after PSO-based threshold optimization and reconstruction exhibit significantly improved discriminative capability. In particular, the proposed method effectively enhances the separability of appliances that share similar electrical characteristics within the same category. As illustrated in Figure 2, four representative household appliances—namely a heater, a hairdryer, a fridge, and a microwave—are selected to compare the original and reconstructed VI trajectory representations.

From the original VI trajectory images, it can be observed that the heater and hairdryer exhibit highly similar patterns, with almost no distinctive features that allow reliable differentiation. After applying the proposed reconstruction method, the trajectory structures undergo noticeable transformations, resulting in clearly distinguishable patterns between the two appliances. A similar phenomenon is observed for the fridge and microwave. Their original VI trajectories present strong visual similarity, which poses challenges for accurate classification. In contrast, the reconstructed trajectories significantly amplify inter-class differences, leading to more distinguishable feature representations. These results demonstrate that the proposed reconstruction strategy effectively mitigates the issue of structural similarity in conventional VI trajectories, thereby providing more discriminative features for subsequent learning-based classification models.

## 3. Multi-Feature Fusion and Optimization Framework

Although the reconstructed VI trajectory enhances the geometric separability of appliances through a threshold-based reflection mapping of voltage and current signals, it still presents several inherent limitations. Specifically, the normalization process applied during VI image construction inevitably weakens amplitude-related information, which is a critical discriminative factor for certain appliance types. Moreover, VI trajectories primarily capture the static phase relationship and geometric patterns between voltage and current, while failing to characterize the dynamic variations of equivalent impedance during appliance operation.

To address these limitations, this study introduces two complementary features: the conductance–time trajectory and the current-squared mean colormap. These features are designed to capture dynamic electrical behavior and global energy characteristics, respectively. A multi-feature fusion framework is further developed to jointly integrate the reconstructed VI trajectory with the proposed auxiliary features, inspired by recent feature-fusion and deep-learning NILM studies [6,27,28].

### 3.1. Conductance–Time Trajectory

Instantaneous conductance provides a direct representation of the internal impedance characteristics of electrical appliances and their temporal variations, complementing VI-trajectory similarity analysis for appliance identification [29]. This is particularly informative for appliances incorporating rectifiers, inductive components, or control modules, whose conductance profiles often exhibit distinct dynamic patterns compared to purely resistive loads such as heating devices. By introducing the conductance–time trajectory, the proposed method supplements the static information contained in VI images with dynamic features, enabling the model to simultaneously learn waveform morphology and time-varying electrical characteristics.

**Figure 3:**
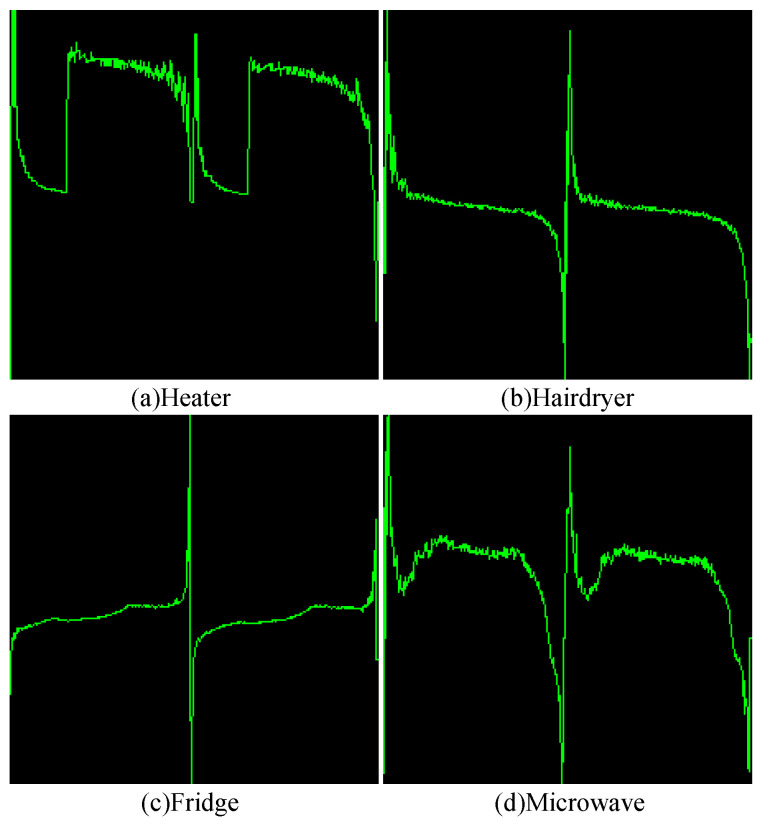
Conductance-time trajectory of several appliances.

Given the acquired steady-state single-cycle voltage and current sequences, denoted as *v* and *i*, the instantaneous conductance *G* at each sampling point is computed as

(9) $$G_{k}=\frac{i_{k}}{v_{k}}$$

where $v_{k}$ and $i_{k}$ represent the voltage and current values at the *k*-th sampling point within one power-frequency cycle, respectively. After obtaining the conductance sequence, the temporal information corresponding to each data point is derived based on the sampling frequency. The time interval between adjacent samples is calculated as

(10) $$T=\frac{1}{F}$$

where *F* denotes the sampling frequency. Accordingly, the timestamp of the *k*-th data point can be expressed as

(11) $$T_{k}=T\times k$$

where *k* is the index of the data point in the sequence. Using the obtained conductance and time sequences, a conductance–time trajectory image is constructed in an RGB color space. Specifically, the conductance sequence is mapped to the vertical axis, while the time sequence is mapped to the horizontal axis. The trajectory is rendered in green (RGB: 0, 255, 0) against a black background (RGB: 0, 0, 0), as illustrated in Figure 3.

From the resulting visualization, it can be observed that high-power appliances tend to produce trajectories concentrated in the upper region of the image, whereas low-power appliances are distributed in the lower regions. Furthermore, appliances with more complex internal structures typically exhibit less smooth trajectories, reflecting richer dynamic variations during operation. These characteristics provide additional discriminative information that is not captured by conventional VI trajectory representations.

### 3.2. Current–Squared Energy Colormap

While the reconstructed VI trajectory and conductance–time features provide rich information in terms of geometric structure and dynamic behavior, they still lack an explicit representation of the magnitude or energy scale of appliance operation. To address this limitation, a current-squared energy colormap is introduced as a third complementary feature. This feature encodes the energy level of each appliance by combining maximum-value normalization, logarithmic compression, and global standardization, thereby enabling a comparable representation across different appliances.

As a result, it effectively compensates for the amplitude insensitivity of VI trajectories and conductance-based features. Even when appliances exhibit highly similar VI shapes or comparable conductance variations, the proposed feature can amplify inter-class differences through energy-level discrimination. To construct this feature, all single-cycle current sequences are first traversed to determine the global maximum current value across the dataset. For each appliance, the mean squared current over a single cycle is computed and normalized as follows:

(12) $$I_{sum}=\frac{\sum_{k=1}^{n}i_{k}^{2}}{n\times i_{\max}^{2}}$$

where $I_{sum}$ denotes the normalized mean squared current for a single cycle, *n* is the number of sampling points, $i_{k}^{2}$ is the current square value at the *k*-th sampling point, and $i_{\max}^{2}$ represents the global maximum square current value across all sequences. The use of squared current values avoids cancellation effects caused by alternating current polarity during summation. However, it also amplifies differences between large and small current values, potentially causing the resulting representation to be dominated by high-amplitude signals while diminishing distinctions among lower-amplitude appliances. To mitigate this issue, a logarithmic normalization is applied

(13) $$I_{sum}^{\prime }=\log\left(1+I_{sum}\right)$$

This transformation effectively compresses the dynamic range and reduces the influence of extreme values. Nevertheless, it may weaken the separability of mid-range values. Therefore, a global standardization step is further introduced

(14) $$\left\{\begin{array}{c} I_{sum}^{\prime \prime }=\frac{I_{sum}^{\prime }-\mu }{\sigma }\\ \mu =\mathrm{mean}(I_{sum}^{\prime })\\ \sigma =\mathrm{std}(I_{sum}^{\prime }) \end{array}\right.$$

where μ and σ denote the mean and standard deviation of $I_{sum}^{\prime }$ over the entire dataset, respectively. This step enhances the discriminability of intermediate values while maintaining robustness against outliers. Finally, the processed feature is linearly mapped to the range [0,255] for image representation

(15) $$b=\frac{I_{sum}^{\prime \prime }-I_{sum,\min}^{\prime \prime }}{I_{sum,\max}^{\prime \prime }-I_{sum,\min}^{\prime \prime }}$$

where *b* denotes the mapped intensity value, and $I_{sum,\min}^{\prime \prime }$ and $I_{sum,\max}^{\prime \prime }$ are the minimum and maximum values of $I_{sum}^{\prime \prime }$, respectively. The resulting values are encoded in the blue channel of an RGB image, forming a pure blue colormap representation (0,0,b).

**Figure 4:**
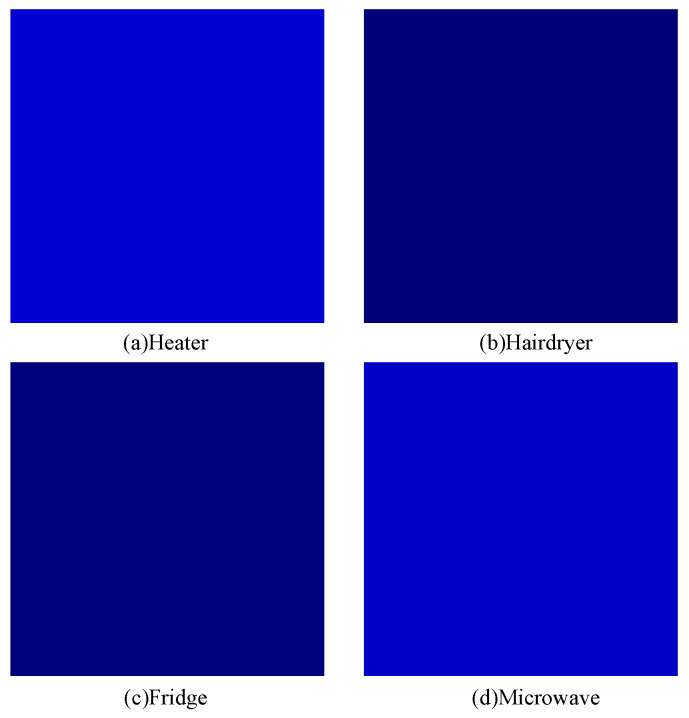
Current-squared energy colormap of several appliances.

As illustrated in Figure 4, it is evident that appliances with different energy levels exhibit distinct blue pixel intensities. Residual CNN appliance-recognition and active-learning NILM studies also indicate the importance of informative feature construction when labeled data are limited [28,30]. This amplitude-based representation effectively compensates for the inherent limitations of reconstructed VI trajectories and conductance–time trajectories in capturing magnitude-related information. Even for appliances exhibiting high geometric similarity in their VI trajectories, the energy colormap amplifies inter-class differences through significant variations in blue channel intensity, thereby providing highly discriminative global energy representations for the subsequent deep convolutional classification network.

### 3.3. Integrated Optimization and Multi–Feature Fusion Network

**Figure 5:**
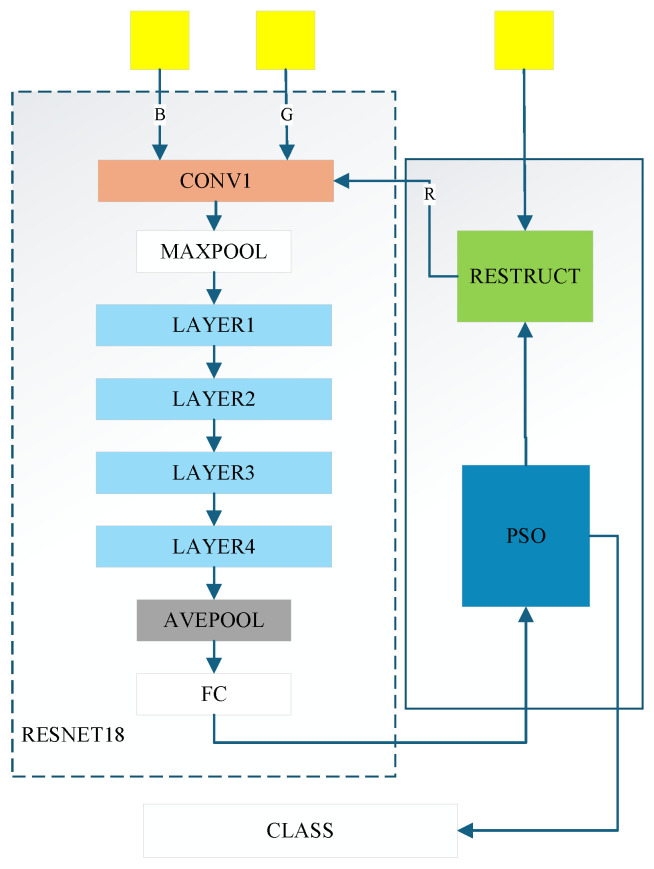
Framework diagram of the integrated optimization and multi-feature fusion network.

In this study, a dual-stage cyclic training framework is developed to jointly integrate threshold optimization, feature reconstruction, multi-feature fusion, and deep learning-based classification, as illustrated in Figure 5. The network design is related to load-trajectory multi-feature learning, ON/OFF event detection, and transformer-based NILM optimization [6,8,31]. The framework consists of three core components: a Particle Swarm Optimization (PSO)-based threshold optimizer, a VI trajectory reconstruction module, and a classification network built upon ResNet18 with an RGB three-channel input scheme. These components are organized into a closed-loop structure, enabling iterative refinement of feature representations and continuous improvement of classification performance.

The resulting feature vector is then fed into a fully connected layer to generate classification outputs. These outputs are further utilized by the PSO-based optimizer to evaluate the fitness of the current threshold configuration. If the fitness criterion is not satisfied, the voltage and current thresholds are updated, and the VI trajectories are reconstructed accordingly. The updated features are then re-encoded and reintroduced into the classification network for the next training iteration. This process continues iteratively until the optimal threshold parameters are obtained, at which point the final classification results are produced.

## 4. Experimental Results and Analysis

### 4.1. Dataset Description

To validate the effectiveness of the proposed method, experiments are conducted on the publicly available PLAID dataset, which contains detailed electrical measurements of appliances from startup to steady-state operation. The dataset includes 1074 instances from 11 appliance categories, with a sampling frequency of 30 kHz [10]. Since current signals are unstable during the transient startup phase, this study focuses on steady-state analysis. Specifically, one cycle of data immediately following a voltage zero-crossing point is selected for feature construction. Given a power frequency of 60 Hz, each cycle contains 500 sampling points. Accordingly, 500 synchronized voltage and current samples are extracted to generate image-based features. Each appliance instance is segmented into multiple steady-state cycles, resulting in 6450 independent samples. All feature generation and model implementation are developed in Python, and the detailed experimental configuration is summarized in Table 1.

To evaluate the generalization capability of the proposed model, the constructed dataset is partitioned into training, validation, and test sets with a ratio of 6:2:2. To ensure that the performance metrics are not biased by unequal class representations across subsets, a stratified sampling strategy is employed during the partitioning process. This guarantees that the proportion of each appliance category remains consistent across all three sets. The detailed sample allocation for each appliance class is presented in Table 2. As observed in the table, the dataset exhibits a mild, naturally occurring class imbalance, with the number of total samples per category ranging from 300 (wash) to 850 (lamp). Such authentic data distributions further validate the robustness of the proposed multi-feature fusion and combinatorial optimization network in handling slightly imbalanced scenarios without the need for synthetic oversampling.

### 4.2. Evaluation Metrics

To comprehensively evaluate the performance of the proposed method, three metrics are employed: classification accuracy, confusion matrix, and F1-score. Accuracy is used to assess overall classification performance and is defined as

(16) $$A_{ccuracy}=\frac{a}{A}$$

where *A* denotes the total number of samples and *a* represents the number of correctly classified samples. The F1-score is adopted to evaluate class-wise performance, defined as the harmonic mean of precision and recall

(17) $$\begin{array}{cc} R_{e} & =\frac{T_{P}}{T_{P}+F_{N}} \end{array}$$

(18) $$\begin{array}{cc} P_{re} & =\frac{T_{P}}{T_{P}+F_{P}} \end{array}$$

(19) $$\begin{array}{cc} F_{1\text{-}score} & =\frac{2\times P_{re}\times R_{e}}{P_{re}+R_{e}} \end{array}$$

(20) $$\begin{array}{cc} F1_{\mathrm{macro}} & =\frac{1}{N}\sum_{i=1}^{N}{F_{1\text{-}score}}_{i} \end{array}$$

where $P_{re}$ and $R_{e}$ denote precision and recall, respectively; TP, FP and FN represent true positives, false positives, and false negatives.

### 4.3. Experimental Results

The constructed dataset is randomly divided into training, validation, and test sets with a ratio of 6:2:2 to evaluate model generalization capability. The training and validation sets are used within the proposed multi-feature fusion and optimization framework. The base classification network is trained for 40 epochs, while the PSO-based optimization process is adaptively iterated according to the defined fitness function until convergence. Related retrainable Siamese networks, weather- and calendar-enhanced disaggregation models, multi-appliance-task learning, EV-oriented NILM, and continual-learning image-signature methods provide methodological context for this experimental design [9,32,33,34,35]. Once the optimal threshold parameters are determined and the reconstructed VI trajectories are finalized, a final training phase is conducted to obtain the optimal model. An adaptive learning rate strategy is adopted, with an initial learning rate of 0.0001. If the loss does not decrease within five consecutive epochs, the learning rate is reduced to one-tenth of its current value. The cross-entropy loss function is employed, and the batch size is set to 32.

**Figure 6:**
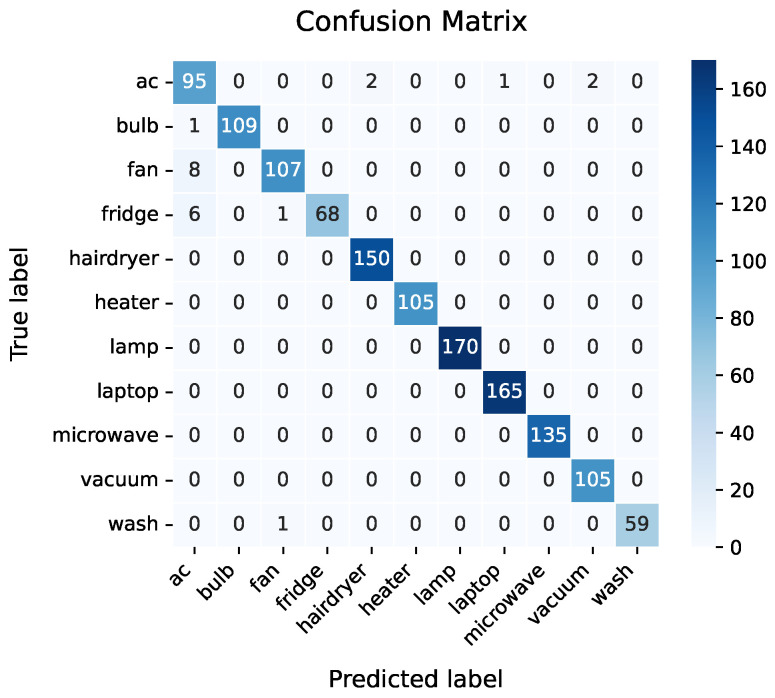
Confusion matrix of test set experimental results.

When evaluated on the test set, the proposed method achieves a classification accuracy of 98.29% on the PLAID dataset. The corresponding confusion matrix is shown in Figure 6, where the horizontal axis represents predicted labels and the vertical axis represents true labels. From the confusion matrix, it can be observed that electric fans and fridges are occasionally misclassified as air conditioners. Meanwhile, air conditioners themselves are prone to misclassification into multiple categories, including hairdryers, computers, and microwaves. These results indicate that multi-state appliances are inherently more difficult to identify due to their diverse operating conditions and highly similar electrical characteristics across different devices.

The $F_{1}$-score for each appliance category is summarized in Table 3. Notably, the air conditioner exhibits a lower $F_{1}$-score compared to other appliances, primarily due to its multiple operating states and its similarity to other appliances under certain conditions. The average $F_{1}$-score reaches 97.93%, demonstrating the overall effectiveness of the proposed method.

To ensure the statistical reliability of the results and mitigate the impact of random initialization, all experiments were independently repeated five times with different random seeds. The results reported in this section represent the average values across these runs. The sample standard deviations for the core metrics (e.g., Accuracy and F-macro) consistently remained within 0.17%, which strongly substantiates the robust stability of the proposed combinatorial optimization network and confirms that the reported performance gains are statistically significant.

### 4.4. Analysis and Discussion

#### 4.4.1. Analysis of Hyperparameter Selection in Fitness Function

To rigorously determine the optimal weighting coefficients (*a*, *b*, *c*) in the PSO fitness function (Equation (8)), a grid search strategy was conducted based on the reconstructed VI trajectory model. The coefficients regulate the trade-off among classification error rate (*a*), cross-entropy loss (*b*), and computational time (*c*). The experimental results are summarized in Table 4.

As shown in the table, when the accuracy weight *a* is set relatively low (e.g., a=0.4,b=0.3,c=0.3), the optimization process under-prioritizes the exact classification decision boundaries, yielding a lower accuracy of 96.46%. Conversely, an excessively high weight on accuracy (e.g., a=0.6,b=0.2,c=0.2) diminishes the regularizing effect of the cross-entropy loss and temporal constraints. This can cause the PSO algorithm to aggressively fit the training accuracy at the expense of generalization and convergence stability, leading to a slight performance degradation (96.94%). The configuration of a=0.5,b=0.3,c=0.2 achieves the highest accuracy (97.05%) by optimally balancing the primary identification task with smooth convergence and reasonable computational overhead.

#### 4.4.2. Effectiveness of Multi-Feature Fusion

Existing studies have shown that models relying solely on VI trajectory features often struggle to accurately distinguish multi-state appliances and appliances operating under similar conditions. The underlying reason is that VI trajectories provide limited electrical information, failing to adequately capture amplitude variations, dynamic impedance changes, and energy-related characteristics. To address this limitation, the proposed multi-feature fusion approach integrates conductance–time trajectories and current-squared energy colormaps to complement the VI representation.

Five groups of experiments are conducted based on the base classification network, and the results are summarized in Table 5. Compared with using only VI trajectories, incorporating the conductance–time feature improves accuracy by 0.24% and ${F1}_{\mathrm{macro}}$ by 0.40%. When both additional features are introduced, the accuracy improves by 0.86% and the ${F1}_{\mathrm{macro}}$ increases by 1.56%. With the VI trajectory included in both configurations, further incorporating the current-squared energy colormap alongside the conductance–time feature increases accuracy by 0.62% and the ${F1}_{\mathrm{macro}}$ by 1.16%. These results demonstrate that the proposed features provide complementary information, effectively enhancing the model’s ability to distinguish between appliances with similar operating characteristics. Furthermore, as demonstrated in the expanded ablation experiments in Table 5, relying solely on the Conductance-Time (CT) trajectory or the Current-Squared Energy (CE) colormap yields suboptimal performance compared to the VI trajectory baseline. The fundamental reason is that while the CT feature effectively captures dynamic impedance and the CE feature highlights global energy amplitude, both intrinsically lack the comprehensive phase mapping and foundational waveform shape information provided by the VI trajectory. Therefore, these auxiliary representations are highly effective as complementary features to amplify differences, but are insufficient to serve as standalone classifiers for complex load identification.

**Figure 7:**
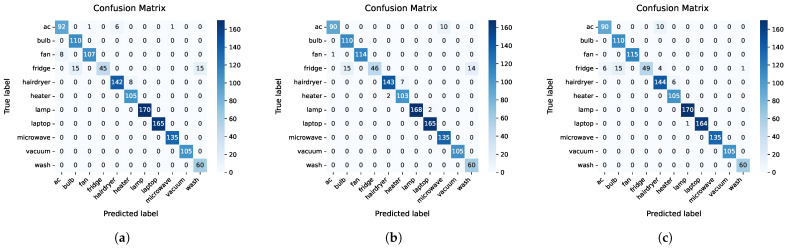
Confusion matrices for different feature combination experiments. (**a**) VI trajectory; (**b**) VI trajectory and conductance–time trajectory; (**c**) three-feature combination.

Further analysis of the confusion matrices (Figure 7) shows that although the recognition rate of air conditioners slightly decreases when combining VI trajectories with conductance–time features, the classification performance for refrigerators, fans, and hair dryers is significantly improved. When all three features are fused, the recognition accuracy of these appliances is further enhanced. This indicates that the proposed features effectively capture dynamic impedance variations and energy-level differences that are not represented in conventional VI trajectories.

#### 4.4.3. Effectiveness of the Integrated Optimization Framework

Although the proposed auxiliary features improve classification performance, the high similarity of VI trajectories among appliances of the same type remains a major limiting factor. To address this issue, the proposed integrated optimization framework introduces adaptive threshold reconstruction via PSO, reducing structural similarity in VI representations.

**Figure 8:**
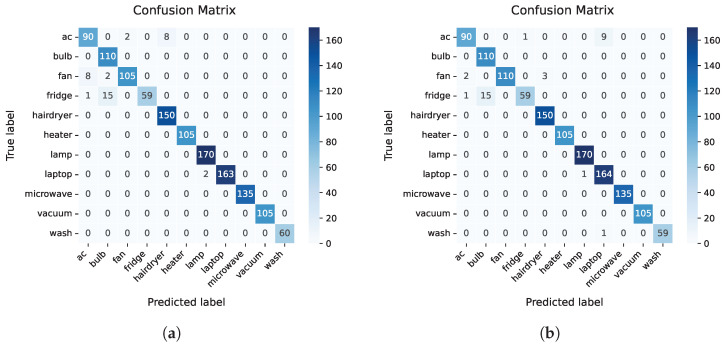
Confusion matrices for the reconstructed VI trajectory and combined feature experiments. (**a**) Reconstructed VI trajectory; (**b**) reconstructed VI and conductance–time trajectories.

Four groups of experiments are conducted to evaluate the effectiveness of the optimization framework, and the results are presented in Table 6 and Figure 8. Compared with original VI trajectories, the reconstructed VI trajectories (Figure 8a) improve classification accuracy by 1.24% and ${F1}_{\mathrm{macro}}$ by 2.32%. When combined with the conductance–time feature (Figure 8b), the reconstructed VI trajectories further enhance performance, yielding improvements of 1.63% in accuracy and 2.73% in ${F1}_{\mathrm{macro}}$. When all three features are integrated, the performance gains reach 2.48% in accuracy and 3.69% in ${F1}_{\mathrm{macro}}$. As shown in Table 6, the inclusion of the original VI trajectory baseline explicitly highlights the critical value of the PSO-based reconstruction module. The original VI trajectories suffer from severe geometric overlap for appliances sharing similar internal topologies, restricting the baseline accuracy to 95.81%. By implementing the adaptive threshold reflection, the reconstructed VI trajectory effectively breaks this structural symmetry, mathematically amplifying the morphological differences between confusing classes. This optimization directly translates to a substantial accuracy leap to 97.05% even without auxiliary features, proving the necessity of the proposed combinatorial optimization.

#### 4.4.4. Comparison with State-of-the-Art Methods

To further substantiate the superiority of the proposed combinatorial optimization network, we compare our results with recently published methods evaluated on the PLAID dataset. A qualitative and quantitative comparison based on published figures from these works is summarized in Table 7.

As shown in Table 7, our method achieves an accuracy of 98.29% and an ${F1}_{\mathrm{macro}}$ of 97.93% across all 11 appliance categories. In comparison, the feature fusion approach combined with SE-ResNet in [27] achieved an accuracy of 96.24%, while the EN-SE-RECNN model relying on 2D load signatures in [28] reached 97.43% accuracy and 97.44% ${F1}_{\mathrm{macro}}$. Our model consistently outperforms these methods, demonstrating that the PSO-based trajectory reconstruction explicitly resolves the geometric overlap of similar loads better than standard multi-channel image mapping.

Although the retrainable Siamese network in [32] reported a slightly higher accuracy of 99.60%, their evaluation was strictly limited to a subset of 10 load categories, excluding highly confusing appliance pairs that degrade overall performance. In contrast, our model maintains robust discrimination across the complete 11-class dataset, representing a more challenging and realistic scenario. Furthermore, compared to the cross-domain SimCLR approach in [15], which achieved a 97.87% ${F1}_{\mathrm{macro}}$ on target domains using 30% labeled data, our method reaches a higher 97.93% ${F1}_{\mathrm{macro}}$ intra-domain by leveraging PSO-optimized multi-feature fusion without the high computational overhead of adversarial domain adaptation. These comparisons firmly substantiate the superiority and competitive edge of the proposed methodology.

## 5. Conclusions

This study addresses the fundamental challenges of appliance load identification by proposing an integrated framework that combines feature optimization, multi-feature fusion, and deep learning-based classification. The main conclusions are summarized as follows:

1. By introducing a PSO-based threshold optimization mechanism and a reflection-based reconstruction strategy, the proposed method preserves the intrinsic electrical characteristics of appliances while amplifying geometric differences among devices with similar structures. This approach effectively overcomes the limitations of conventional VI trajectories, where high structural similarity often leads to classification ambiguity, thereby providing more discriminative feature representations for downstream tasks.
2. The conductance–time trajectory captures dynamic impedance variations during appliance operation, while the current-squared energy colormap encodes global energy-level differences. These features complement the reconstructed VI trajectory by incorporating dynamic and amplitude-related information. Experimental results demonstrate that the combination of all three features improves classification accuracy by 0.86% and the ${F1}_{\mathrm{macro}}$ by 1.56% compared to using VI trajectories alone, effectively addressing the issues of weakened amplitude information and missing dynamic characteristics.
3. The proposed closed-loop architecture, integrating PSO-based threshold optimization with a ResNet18 classification network, enables adaptive adjustment of reconstruction parameters. This ensures that the model is consistently trained with near-optimal feature inputs. The framework achieves a classification accuracy of 98.29%, outperforming the baseline model without optimization. Notably, within the evaluation scope of the PLAID dataset, the proposed method demonstrates improved recognition performance for certain multi-state appliances, such as air conditioners and refrigerators, compared to baseline models, mitigating the impact of high intra-class variability.
4. Validation on the publicly available PLAID dataset shows that the method achieves robust performance across 11 common household appliance categories, with an ${F1}_{\mathrm{macro}}$ of 97.93%. Furthermore, the proposed framework is computationally feasible and can be readily deployed, making it a promising solution for real-world applications such as residential energy monitoring and smart grid load management.

Despite the promising results, this study has limitations regarding zero-shot generalization to completely unknown electrical equipment and unseen operating conditions. Therefore, future work will focus on integrating continual learning and open-set recognition mechanisms to adaptively handle new appliance signatures without retraining from scratch. Furthermore, we aim to expand the diversity of appliance types in the dataset and improve the adaptive parameter tuning strategy of the PSO algorithm, ultimately enhancing the robustness and scalability of the proposed method in complex, heterogeneous smart grid scenarios.

## Figures and Tables

**Table 1:** Experimental environment parameters.

Hardware/Software	Version/Model
Operating System	Windows 11 (64-bit)
CPU	Intel(R) Core(TM) i5-12600KF
GPU	RTX 4060
Python	3.9
PyTorch	2.7.1
CUDA	11.8

**Table 2:** Sample distribution of the dataset across training, validation, and test sets.

Appliance Class	Training Set (60%)	Validation Set (20%)	Test Set (20%)	Total (100%)
ac	300	100	100	500
bulb	330	110	110	550
fan	345	115	115	575
fridge	225	75	75	375
hairdryer	450	150	150	750
heater	315	105	105	525
lamp	510	170	170	850
laptop	495	165	165	825
microwave	405	135	135	675
vacuum	315	105	105	525
wash	180	60	60	300
Total	3870	1290	1290	6450

**Table 3:** $F_{1}$ score table for 11 load types.

Load	$F_{1}$ Score/%	Load	$F_{1}$ Score/%
Ac	90.48%	Lamp	99.71%
Bulb	99.54%	Laptop	100.00%
Fan	95.96%	Microwave	99.26%
Fridge	93.15%	Vacuum	100.00%
Hairdryer	100%	Wash	99.16%
Heater	100%	$F_{\mathrm{macro}}$	97.93%

**Table 4:** Grid search results for weighting coefficients in the fitness function.

*a* (Error Rate)	*b* (Loss)	*c* (Time)	Accuracy/%
0.4	0.3	0.3	96.46
0.5	0.3	0.2	97.05
0.6	0.2	0.2	96.94

**Table 5:** Table of accuracy and ${F1}_{\mathrm{macro}}$ score for feature combination experiments.

Feature	Accuracy/%	${F1}_{\mathrm{macro}}$/%
VI Trajectory	95.81%	94.24%
Conductance–Time Trajectory	85.89%	84.08%
Current-Squared Energy	37.98%	30.29%
VI Trajectory + Conductance–Time Trajectory	96.05%	94.58%
VI Trajectory + Conductance–Time Trajectory + Current-Squared Energy Colormap	96.67%	95.80%

**Table 6:** Experimental results of different feature combinations in combinatorial optimization networks.

VI Trajectory	Conductance–Time Trajectory	Current-Squared Energy Colormap	Accuracy/%	${F1}_{\mathrm{macro}}$/%
Original			95.81%	94.24%
Reconstructed			97.05%	96.56%
Reconstructed	✓		97.44%	96.97%
Reconstructed	✓	✓	98.29%	97.93%

**Table 7:** Comparison with state-of-the-art methods on the PLAID dataset.

Method	Reference	Evaluated Classes	Accuracy/%	${F1}_{\mathrm{macro}}$/%
Feature Fusion + SE-ResNet	[27]	11	96.24%	-
2D Signatures + EN-SE-RECNN	[28]	11	97.43%	97.44%
SimCLR + Color V-I (Cross-domain)	[15]	11	-	97.87%
Retrainable Siamese Network	[32]	10	99.60%	99.20%
Proposed Method	Ours	11	98.29%	97.93%

## Data Availability

Publicly available datasets were analyzed in this study.
